# Supplementary material for: Role and mechanism of NCAPD3 in promoting malignant behaviors in gastric cancer
Source: Front Pharmacol. 2024 Apr 22;15:1341039. doi: 10.3389/fphar.2024.1341039 (PMC11070777; doi:10.3389/fphar.2024.1341039)
Supplement: Supplementary file 11 [file DataSheet2.ZIP › GSEA/Oncogenic signature/my_analysis.Gsea.1599461962066/KRAS.600.LUNG.BREAST_UP.V1_UP.html]

Details for gene set KRAS.600.LUNG.BREAST\_UP.V1\_UP[GSEA]

|  || Dataset | filtered\_dataset.sample\_info.cls#WT\_versus\_NCAPD3\_MUT |
| Phenotype | sample\_info.cls#WT\_versus\_NCAPD3\_MUT |
| Upregulated in class | NCAPD3\_MUT |
| GeneSet | KRAS.600.LUNG.BREAST\_UP.V1\_UP |
| Enrichment Score (ES) | -0.40083262 |
| Normalized Enrichment Score (NES) | -1.9085969 |
| Nominal p-value | 0.009280742 |
| FDR q-value | 0.03178736 |
| FWER p-Value | 0.244 |
Table: GSEA Results Summary

  

Fig 1: Enrichment plot: KRAS.600.LUNG.BREAST\_UP.V1\_UP      
 Profile of the Running ES Score & Positions of GeneSet Members on the Rank Ordered List

  

| SYMBOL | TITLE | RANK IN GENE LIST | RANK METRIC SCORE | RUNNING ES | CORE ENRICHMENT || 1 | 160518 | DENND5B | 13 | 1.060 | 0.0649 | No |
| 2 | 2921 | CXCL3 | 57 | 0.884 | 0.0959 | No |
| 3 | 26272 | FBXO4 | 133 | 0.767 | 0.0957 | No |
| 4 | 3770 | KCNJ14 | 157 | 0.721 | 0.1297 | No |
| 5 | 2919 | CXCL1 | 405 | 0.534 | -0.0106 | No |
| 6 | 2920 | CXCL2 | 454 | 0.501 | -0.0100 | No |
| 7 | 3673 | ITGA2 | 858 | -0.264 | -0.2814 | No |
| 8 | 5329 | PLAUR | 952 | -0.373 | -0.3222 | No |
| 9 | 29948 | OSGIN1 | 1002 | -0.406 | -0.3291 | No |
| 10 | 3669 | ISG20 | 1097 | -0.475 | -0.3634 | No |
| 11 | 3706 | ITPKA | 1150 | -0.514 | -0.3648 | Yes |
| 12 | 639 | PRDM1 | 1159 | -0.523 | -0.3339 | Yes |
| 13 | 7128 | TNFAIP3 | 1198 | -0.573 | -0.3211 | Yes |
| 14 | 6690 | SPINK1 | 1200 | -0.577 | -0.2814 | Yes |
| 15 | 330 | BIRC3 | 1224 | -0.600 | -0.2560 | Yes |
| 16 | 50856 | CLEC4A | 1249 | -0.630 | -0.2291 | Yes |
| 17 | 1839 | HBEGF | 1328 | -0.751 | -0.2326 | Yes |
| 18 | 1649 | DDIT3 | 1341 | -0.779 | -0.1866 | Yes |
| 19 | 9966 | TNFSF15 | 1376 | -0.900 | -0.1480 | Yes |
| 20 | 7462 | LAT2 | 1399 | -1.138 | -0.0842 | Yes |
| 21 | 4312 | MMP1 | 1407 | -1.304 | 0.0022 | Yes |
Table: GSEA details [plain text format]

  

Fig 2: KRAS.600.LUNG.BREAST\_UP.V1\_UP      
 Blue-Pink O' Gram in the Space of the Analyzed GeneSet

  

Fig 3: KRAS.600.LUNG.BREAST\_UP.V1\_UP: Random ES distribution      
 Gene set null distribution of ES for **KRAS.600.LUNG.BREAST\_UP.V1\_UP**
